# A Novel Set of WNT Pathway Effectors as a Predictive Marker of Uterine Corpus Endometrial Carcinoma–Study Based on Weighted Co-expression Matrices

**DOI:** 10.3389/fonc.2019.00360

**Published:** 2019-05-10

**Authors:** Katarzyna Kośla, Magdalena Orzechowska, Dorota Jędroszka, Izabela Baryła, Andrzej K. Bednarek, Elżbieta Płuciennik

**Affiliations:** Department of Molecular Carcinogenesis, Medical University of Łódz, Łódz, Poland

**Keywords:** Wnt signalling pathway, endometrioid carcinoma, prognosis, co-expression matrices, ROC

## Abstract

Uterine corpus endometrial carcinomas (UCEC) are clinically divided into two subgroups—endometrioid endometrial carcinoma (EEC) or non-endometrioid endometrial carcinoma (NEEC). The first group shows relatively better prognosis. However, the discrimination seems to be insufficient due to the fact that in the mildest EEC are patients with poor treatment response and bad prognosis. Our aim was to examine the molecular background of such phenomenon and whether gene expression patterns might be of importance for the clinic. We focused our analysis on WNT pathway target genes since it is one of the main regulators of endometrial proliferation and differentiation. *In silico* analysis of TCGA data, including Weighted Co-expression Network Analysis, Principle Component Analysis, and Multiple Factor Analysis, allows to select 28 genes that serve as a predictive markers for UCEC patients. Our study revealed that there is a subgroup of the endometrioid cases that molecularly resembles mixed/serous groups. This may explain the reason for existence of subgroup of patients, that although clinically diagnosed with the mildest endometrioid UCEC type, yet present failure in treatment and aggressive course of the disease. Our study suggests that worse outcome in these patients may be based on a disruption of proper WNT signalling pathway resulting in deregulation of its effector genes. Moreover, we showed that mixed group consisting of tumours containing both endometrioid and serous types of cells, has serous expression profile of WNT targets. The proposed gene set allows to predict progression of the disease trough dividing patients into groups of low or high grade with 70.8% sensitivity and 88.6% specificity (AUC = 0.837) as well as could predict patient prognosis associated with UCEC subtype with 70.1% sensitivity and 86.2% specificity (AUC = 0.855). Relatively small number of implicated genes makes it highly applicable and possibly clinically simple and useful tool.

## Introduction

Uterine Corpus Endometrial Carcinoma (UCEC) is one of the most common gynaecological cancers which can spread outside the uterus through myometrium invasion (1). Although most of UCEC cases are low grade and have a favourable prognosis, there is a subgroup of highly malignant tumours which accounts for high mortality rate.

In 1983 Bokhman et al. demonstrated two distinct pathways of uterine malignancies development. Endometrioid endometrial carcinoma (EEC) represents about 70–80% of all UCEC cases and is observed in young pre- and post-menopausal women. EEC is typified by expression of oestrogen (ER) and progesterone (PR) receptors, so it is thought to be influenced by endocrine modulation (oestrogen unopposed by progesterone) and follows the oestrogen-related pathway (2, 3). Non-endometrioid endometrial carcinoma (NEEC) is diagnosed in relatively older, postmenopausal women, in the background of an atrophic endometrium without association with oestrogen stimulation. NEECs are high-grade tumours characterized by serous papillary or clear-cell morphology, as well as aggressive clinical course and poor prognosis, resulting from high potential for deep myometrial invasion and lymphatic spread. NEEC tumours are characterized by poor differentiation and high risk of deep myometrial invasion (2–6).

In 2013, the Cancer Genome Atlas (TCGA) Research Network published the molecular classification based on the analysis of copy number, microsatellite instability, genome sequencing, and exome sequence. It divides UCEC tumours into four group: POLE ultramutated, MSI hypermutated, copy-number (CN) low, and CN high presenting different risk for disease progression (7–9).

In these molecular based groups, a significant histopathological diversity is observed. For example, the best prognosing POLE group surprisingly contains both NEEC and high grade EEC phenotypes but with good prognosis (10). This indicates the need for research in order to search for molecular classifiers for better prediction of the course of the disease.

We decided to use an integrated expression analysis to find groups of genes correlated with specific prognosis. Our goal was to create a relatively small set of genes whose expression would predict the patients' prognosis independently of the grade and histological type. We focused on WNT signalling pathway as one of the pivotal intracellular regulator.

Wnt/β-catenin signalling pathway is deregulated in 40% of endometrial cancers. This signalling track is essential for maintaining balance in the proliferation and differentiation of endometrium through sex hormone regulation during the menstrual cycle (11–13).

Contrary to most of the studies, we concentrated on the downstream WNT effector proteins whose expression is regulated by TCF/LEF transcription factors.

## Results

### Downstream Effects of Wnt Signalling in UCEC

As Wnt alterations affect aggressiveness of UCEC we focused on downstream effects of the pathway through consideration of 2,574 target genes of Wnt–specific transcription factors (TCF/LEF family).

### Alterations in Functional Networks of Wnt Downstream Effectors Reveal Patient Prognosis Independently of Histological Type of UCEC–Weighted Gene Co-expression Network Analysis (WGCNA)

**Figure 1:**
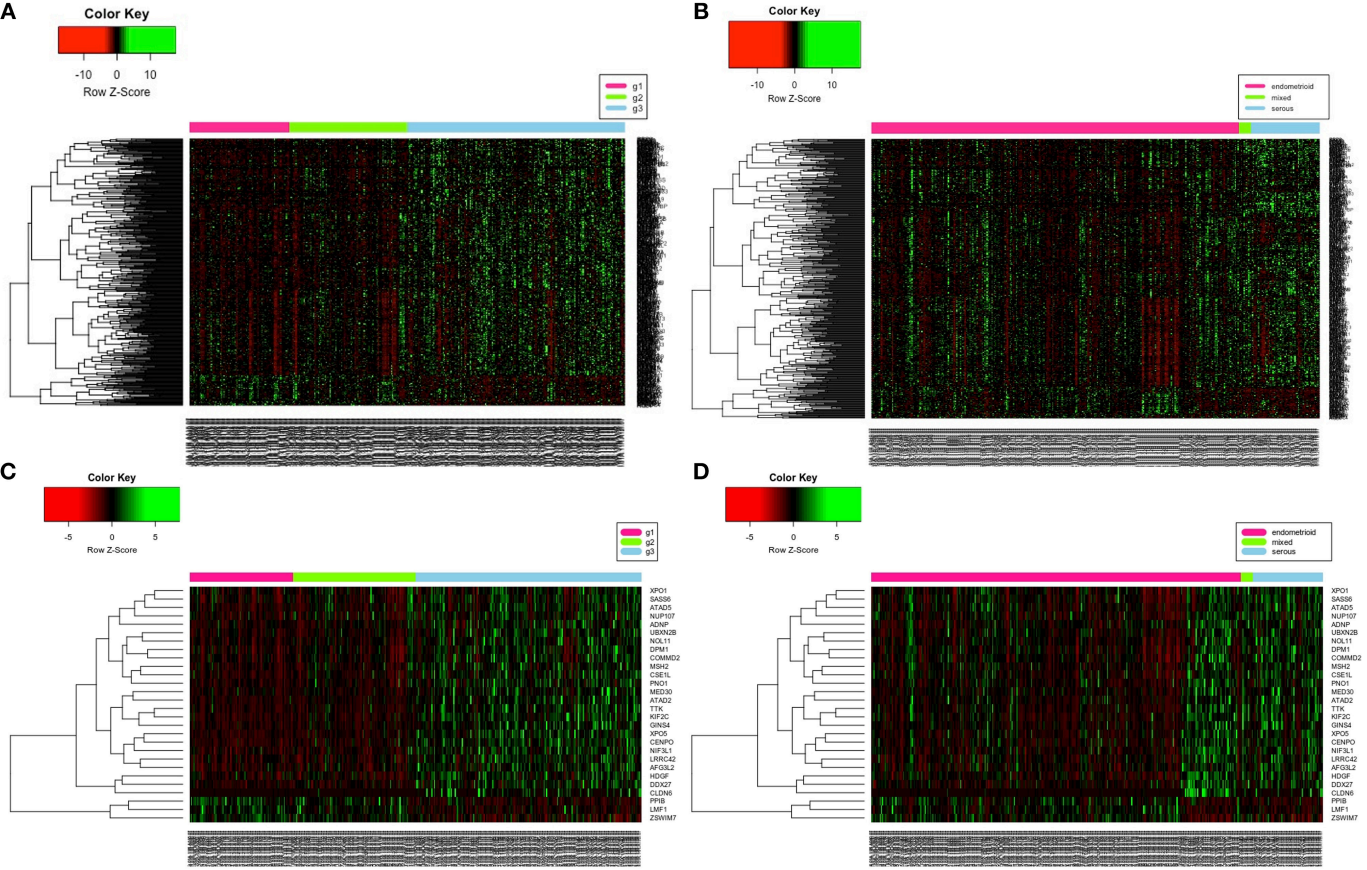
**(A,B)** Heatmaps reflect expression of 492 genes belonging to turquoise module according to UCEC grade and histology, respectively, whereas **(C,D)** heatmaps reflect expression of selected set of genes that differentiates grade 1 and 2 from grade 3 cases **(C)** and highlight existence of novel molecular subgroup of endometrioid endometrial adenocarcinomas more similar to serous subtype **(D)**.

**Figure 2:**
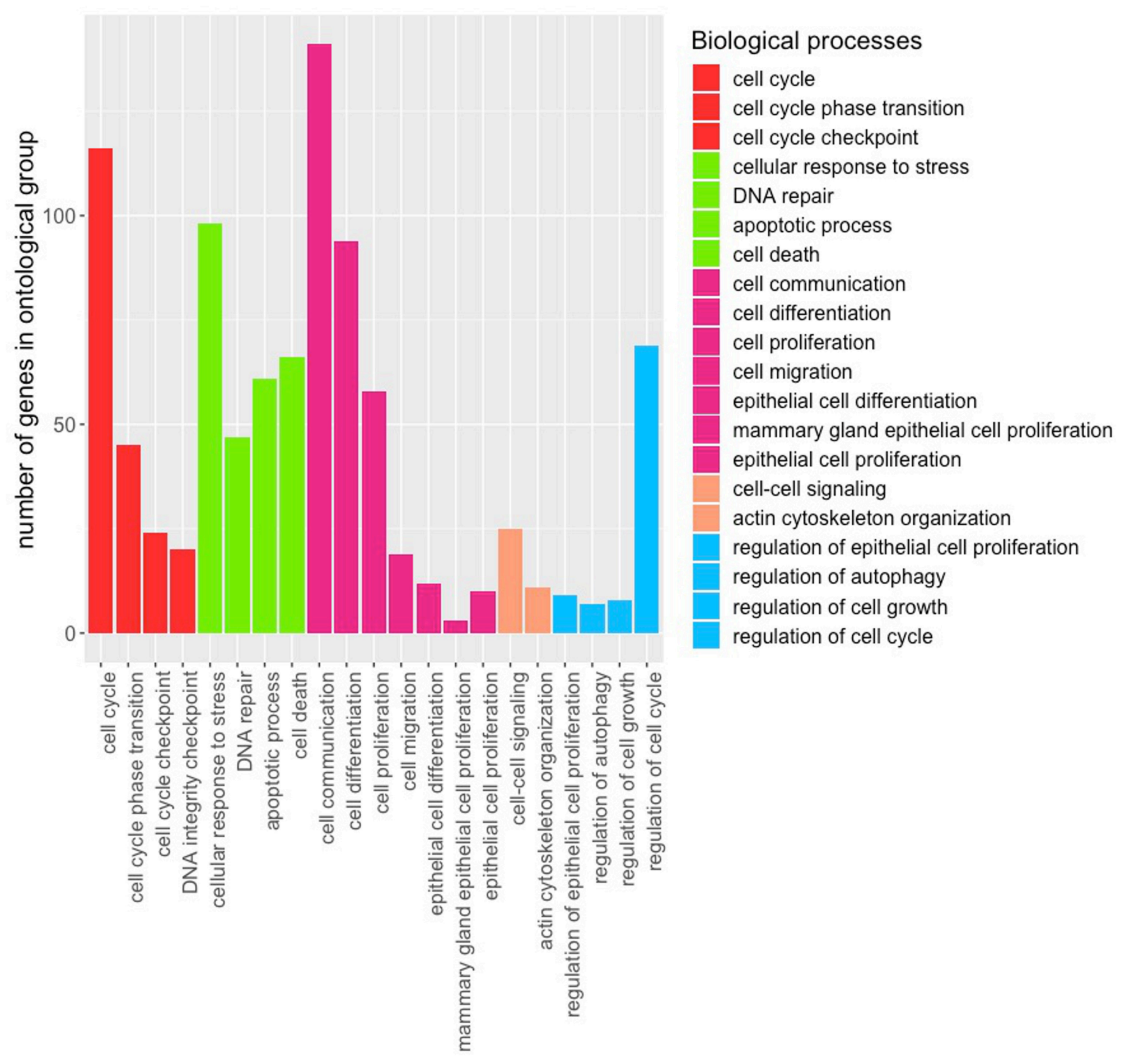
Barplot presenting biological ontology of turquoise module.

This study aimed to identify molecular signatures within Wnt endpoint effects that may be associated with histological subtypes and grade of UCEC. Hereby applied WGCNA algorithm transforms the adjacency matrix into the distance matrix of co-expression, based on topological overlap, using in this study soft thresholding power of seven followed by gene hierarchical clustering and dynamic tree-cutting algorithm. This algorithm reveals clusters of genes (hereinafter referred to as modules) of a common expression profile in correlation with clinical traits of UCEC. Thus, it was possible to define nine distinct modules of different sizes (black: 50 genes, blue: 442 genes, brown: 183 genes, green: 107 genes, pink: 39 genes, red: 60 genes, turquoise: 492 genes, yellow: 147 genes, grey: 1053 genes) that were correlated with clinical traits such as histological subtype and grade. The most numerous grey module was discarded from further analyses as it includes all genes that do not share any pattern of expression. Supplementary Figure 1 presents the topological overlap matrix accompanied by hierarchical clustering dendrograms and module colours (referred as TOM plot), which indicates genes of high connectivity that tend to be essential for studied clinical traits. MEs considered as the most representative gene expression profiles were calculated for each module as the first principle component of the standardized expression profiles within a particular module to identify sets of genes reflecting tumour histology or grade in a significant manner. According to the module-trait relationship plot we found turquoise and yellow modules showing the highest positive correlation with both clinical traits (histology: *r* = 0.23, *p* = 6e−6, *r* = 0.38, *p* = 5e−14, respectively, and grade: *r* = 0.44, *p* = 3e−19, *r* = 0.22, *p* = 2e−05, respectively) (Supplementary Figure 2). Both of them were also of the highest GS regarding UCEC grade (Supplementary Figure 3A) and histology (Supplementary Figure 3B), however the yellow module has been eventually omitted due to insufficient association between module membership (MM), defined as correlation metric of gene expression profile with ME, and GS (Supplementary Figure 4). Finally, the expression profiles of 492 turquoise module genes were visualized in a form of heatmaps and noteworthy, all of them showed two types of visible associations: firstly, molecular differentiation between UCEC grades (Figure 1A) and secondly, molecular differentiation between histological subtypes revealing a novel subgroup of UCEC patients consisting of endometrioid, mixed and serous histologies (Figure 1B). In order to associate the genes of turquoise module with biological processes that they might be involved in, enrichment analysis within GO BP was performed. Among enriched terms, several important findings emerged indicating potential causes of clinical differences between identified subtypes of UCEC or grades that may stem from alterations in the cell cycle, proliferative and differentiation processes as well as cellular death and DNA repair processes (Figure 2).

### Dimensional Grouping of UCEC Patients According to Identified Molecular Profiles Associating Histology and Grade—Principle Component Analysis (PCA) and Multiple Factor Analysis (MFA)

**Figure 3:**
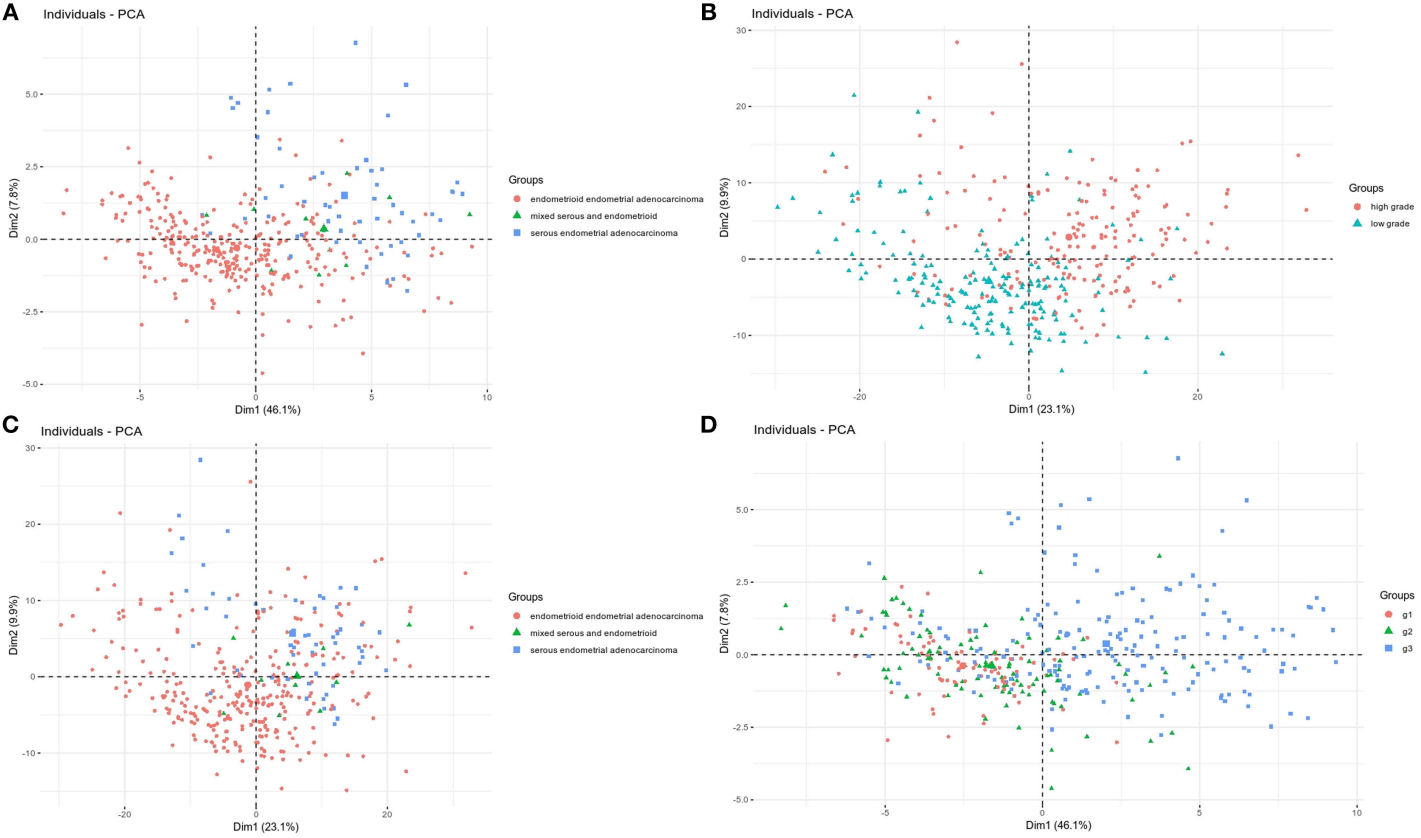
Turquoise module genes show partitioning of UCEC cases of **(A)** low vs. high grade with a total variance of 33% and **(B)** endometrioid endometrial adenocarcinoma vs. mixed and serous endometrial adenocarcinoma with a total variance of 33%. Set of selected 28 genes **(C)** clearly partition grade 3 cases from mixed group of grade 1 and grade 2 and **(D)** partition in majority cases endometrioid endometrial adenocarcinoma from mixed and serous subtypes.

We employed PCA to partition UCEC patients within two principle components according to the molecular profile of 492 turquoise module genes in association with both, histology and grade. We found clear partitioning of individuals across the first dimension with a total variance of 33% (Figures 3A,B). PCA was followed by MFA, which served to identify genes of the highest contribution to spatial partitioning of patients and select genes differentiating clinical groups in the most significant manner. This resulted in selection of 28 variables that differentiated individuals across the first principle component to the extent of 46.1% of the total variance that reflected molecular differences between low and high grades, and simultaneously between endometrioid and mixed groups of endometrioid, mixed and serous cancer subtypes (Figures 1C,D, 3C,D).

### Selected Effectors of Wnt Pathway May Serve as Predictive Markers of UCEC Prognosis—Binomial Model of Logistic Regression and Receiver Operating Characteristic (ROC) Curve

**Figure 4:**
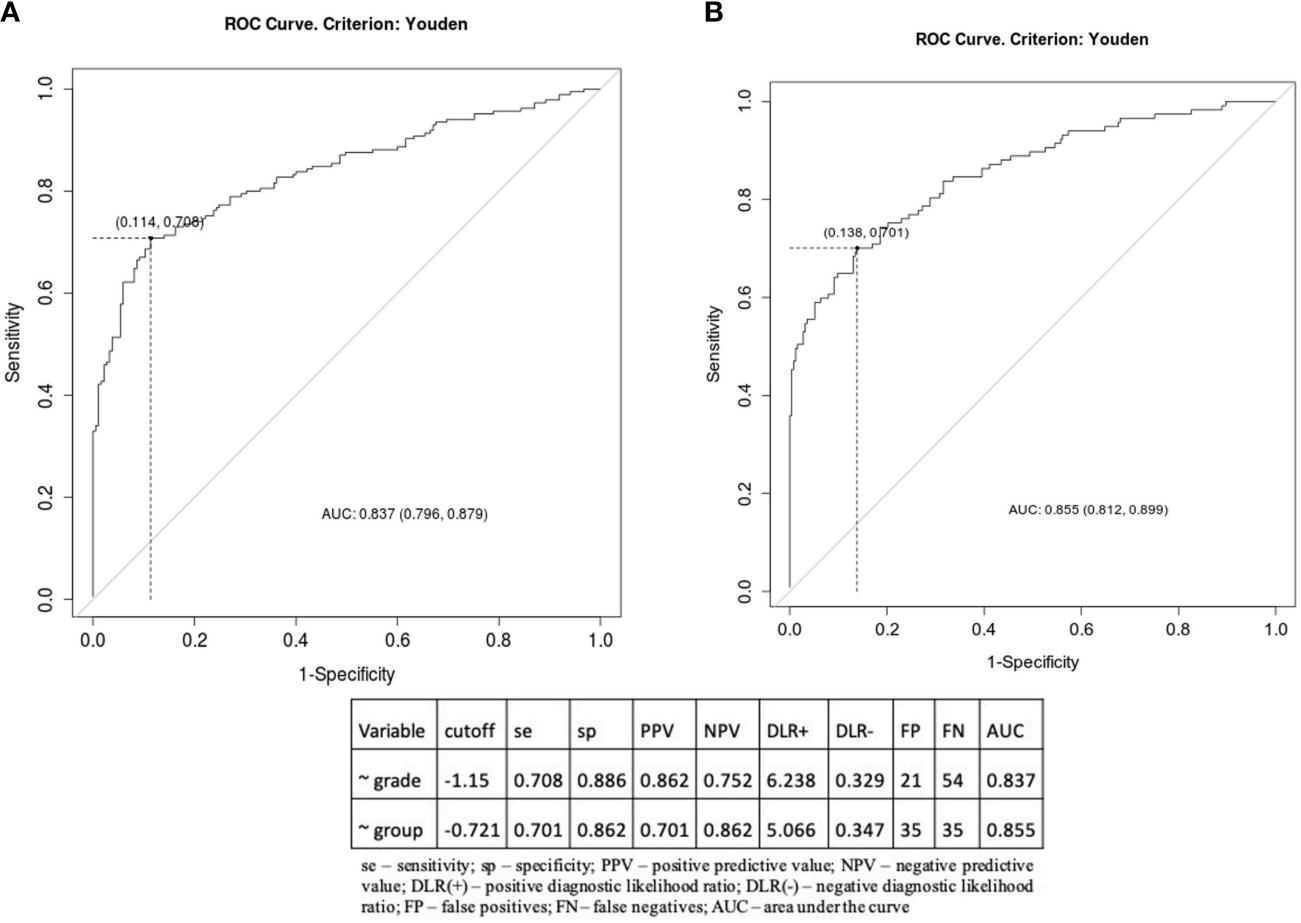
ROC curve according to **(A)** grade, **(B)** patient prognosis with detailed statistics.

**Figure 5:**
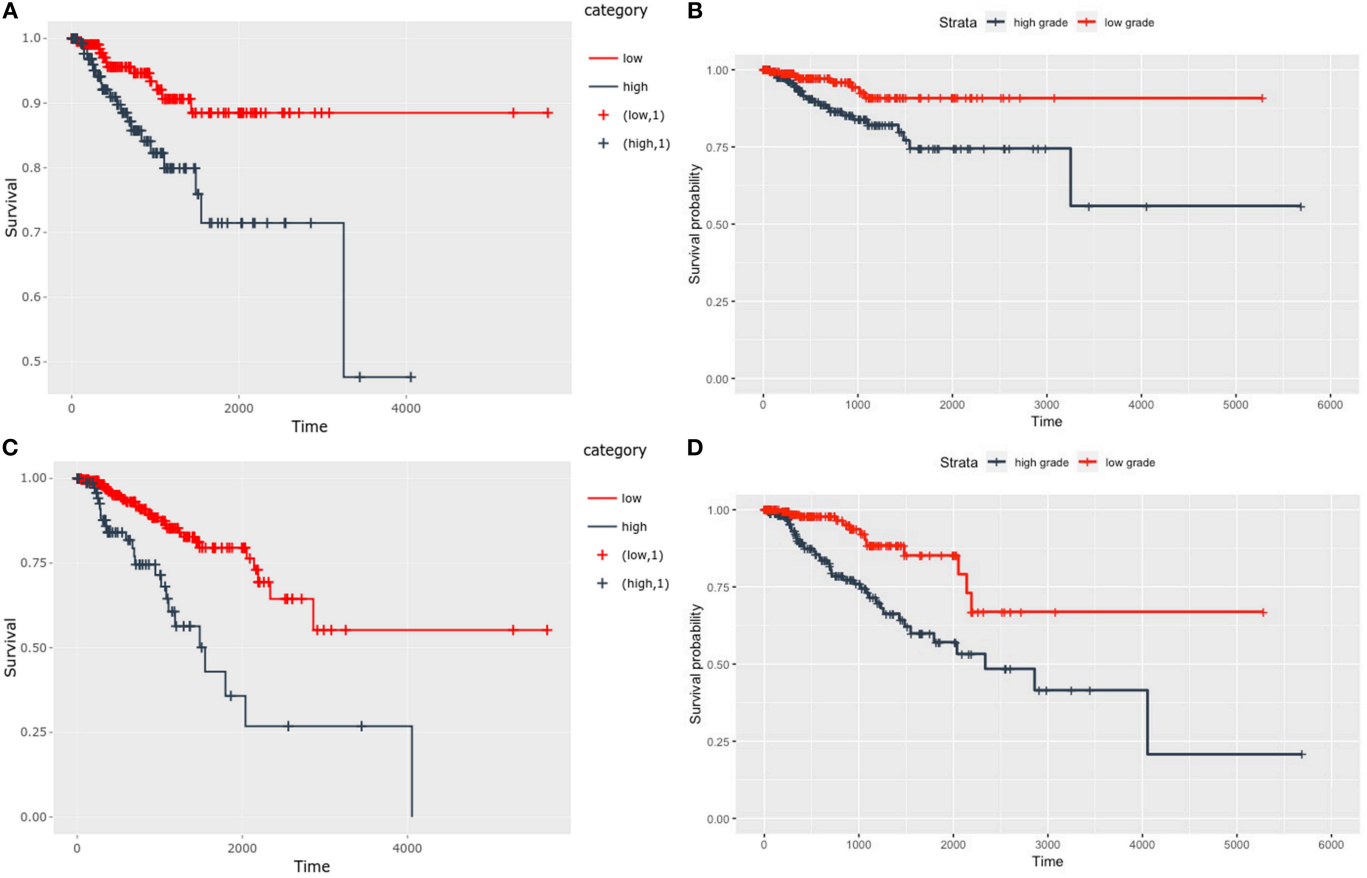
Kaplan-Meier plots comparing predictive potential of presented marker and standard classification by grade for **(A–B)** overall survival and **(C–D)** disease-free survival, respectively.

We developed a predictive marker through the binomial model of logistic regression based on the combined expression of selected 28 genes in association with a defined molecular subtype. According to ROC, the developed marker could predict a level of UCEC progression through dividing patients into groups of low or high grade with 70.8% sensitivity and 88.6% specificity (AUC = 0.837) as well as could likewise predict patient prognosis associated with UCEC subtype with 70.1% sensitivity and 86.2% specificity (AUC = 0.855) (Figure 4). Moreover, the prognostic potential of presented marker has been confirmed with Kaplan-Meier plots for both, overall survival (OS), and disease-free survival (DFS). Interestingly, the split of UCEC patients was of much higher accuracy in comparison to standard OS and DFS according to grade (Figure 5). Detailed statistics are shown in Table 1.

**Table 1:** Detailed statistics of survival analysis comparing novel marker with standard grade classification.

**Prediction according to**	**Cutpoint**		**HR**		***p*****-value**	
	**OS**	**DFS**	**OS**	**DFS**	**OS**	**DFS**
28 geneset marker grade	−0.9918	−0.1172	88	199	4.13e−95	4.84e−108
	not applicable		0.35	0.32	0.0078	0.00013

### Functional Network of Wnt Effectors Reveals Biological Differences Between Novel UCEC Subtypes—Network Analyst

**Figure 6:**
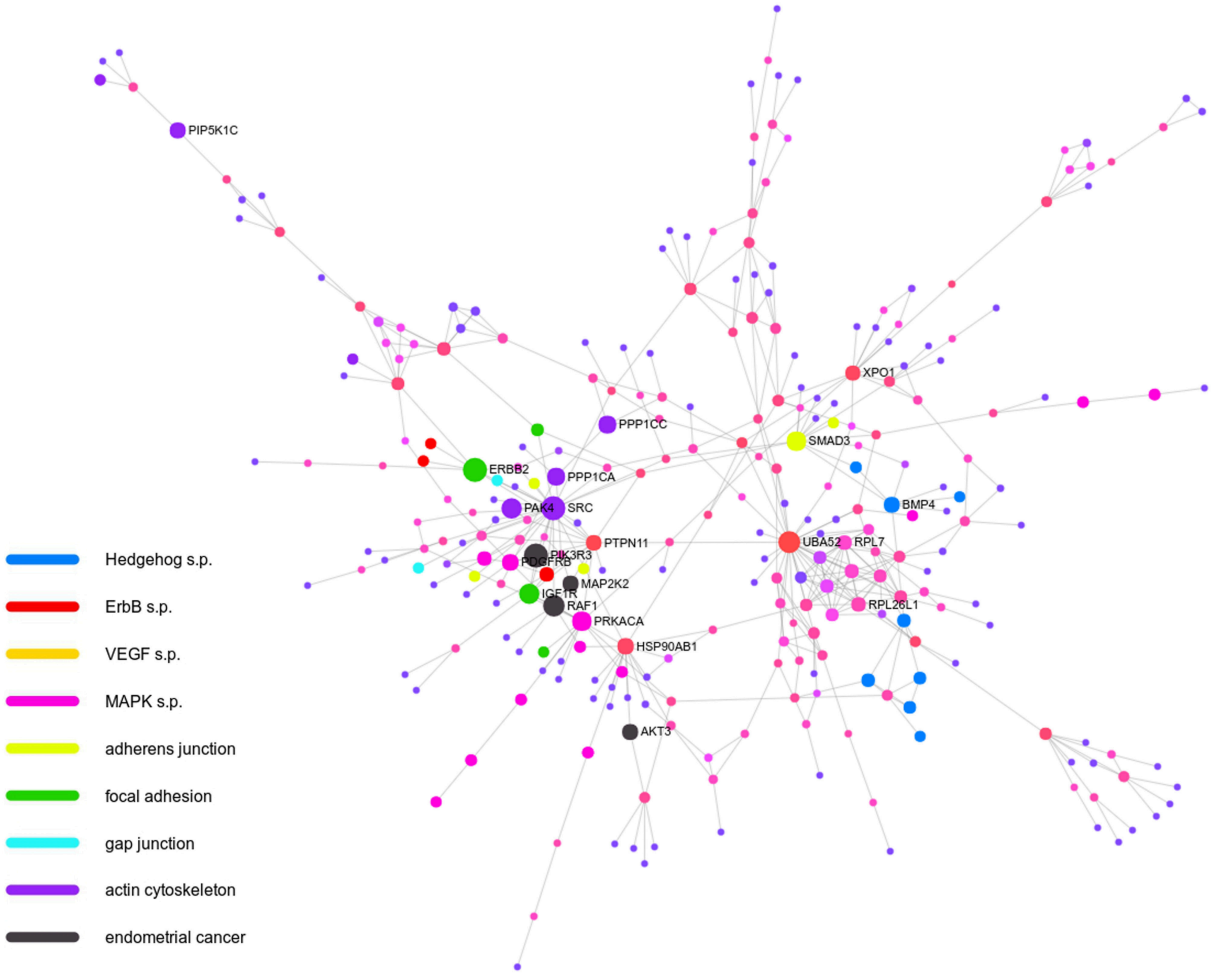
Global functional network differentiating molecular subgroups of UCEC coloured according to biological process.

Finally, we performed the analysis of functional networks within downstream effectors of Wnt that were altered in molecular subtypes of UCEC to identify main biological processes involved in increased UCEC aggressiveness. Global network was constructed with 286 nodes and 455 edges (FDR < 0.05) that revealed overrepresentation of several pathways and biological processes essential in malignant carcinomas. In the network, we identified 9 genes involved in Hedgehog signalling pathway, 10 genes of ErbB signalling, 7 genes associated with VEGF pathway, and 15 genes involved in MAPK cascade. Moreover, many adhesion-associated processes were enriched such as the adherens junction, focal adhesion, gap junction, and regulation of actin cytoskeleton that are known to accelerate tumour progression and invasiveness. Noteworthy, the molecular signature of endometrial cancer was likewise identified (Figure 6).

### Significance of Mutations/CNVs in Wnt Pathway Members

Of 42 major Wnt pathway players we found only 5 of high significance regarding mutations and CNVs (Supplementary Table 1). They varied in number of altered cases between novel molecular subgroups of UCEC; specifically, *APC, CTNNB1B, LRP5P*, and *TCF4F* were found mutated mostly in group of endometrioid endometrial cancer cases and *MYC* was the only gene of altered copy number with a predominance in group of endometrioid, mixed and serous endometrial cancer cases.

### Validation of the Results

Cross—validation of the findings based on independent UCEC cohorts was not performed due to lack of relevant expression data in association with specific clinical information.

## Discussion

Endometrial cancer is one of the most common gynaecological cancers with morbidity rates tending to increase (14). The majority of endometrial tumours present low stage and grade with favourable prognosis, although some fractions of patients experience different course of disease and significantly worse outcome (15). Nowadays, there are two main classification systems of endometrial cancer. The former created by Bokhman et al. is a historical classification based on the epidemiological, clinical and endocrine relationship that divides UCEC into two types: estrogen-related endometrioid endometrial carcinoma (EEC) and non-endometrioid endometrial carcinoma (NEEC). However, this division does not reflect the real nature of the endometrial malignant pathology and does not include clear-cell carcinoma, carcinosarcomas or other undifferentiated carcinomas (4). Since 2013, a new, TCGA-based, molecular classification of endometrial cancer has been provided consisting of four genomic groups that vary in clinical outcome. The group with an increased number of gene copies (CN high) was characterized by the lowest survival rate (survivability at 50% after 5 and 10 years); the best-performing group was POLE (survivability at 100%), while the MSI hypermutated group and the CN low group had a similar survival rate of 80% (8, 9). Interestingly, the molecular and classical Bokhman's classification systems do not overlap; for example, EEC grade 3 cancers do not constitute a separate molecular subgroup, since they are randomly distributed across all four TCGA-based groups (7).

In our study, we employed WGCNA approach to identify genetic profiles associated with specific unfavourable prognosis of UCEC that could biologically explain such phenomenon. Our goal was to determine a relatively small set of genes that could be used to predict patients' prognosis independently of grade and histological type. We focused on the Wnt signalling pathway as one of the pivotal intracellular regulators of endometrium proliferation and differentiation, very often deregulated in endometrial adenocarcinoma.

In fact, alterations in the Wnt pathway are commonly observed in endometrial cancer at different signalling levels, e.g., ligands, main effector- β-catenin, APC and Axins that affect executive transcription factors followed by deregulation of downstream effector target genes (11–13, 16–18). Therefore, unlike most of the reported studies, we decided to analyse downstream effects of Wnt disruption. The analysis was conducted on TCGA data collected from 370 UCEC patients. We identified a total of nine co-expression modules; primarily two of them were correlated with clinical characteristics such as grade and histological type of UCEC, although eventually the turquoise module was of the highest significance (cor > 0.4, *p* = 3e−19) that corresponded to 492 differentially expressed Wnt target genes (Supplementary Figure 3B). GO analysis revealed that they were mainly involved in the cell cycle, response to cellular stress and DNA damage, differentiation and proliferative processes, cell architecture and communication, as well as regulation (Figure 2). Noteworthy, these findings showed key alterations resulting from the disrupted signalling via Wnt pathway that may modify patients' prognosis due to the molecular profile of more aggressive features. Moreover, deregulation of these processes clearly refers to hallmarks of cancer such as acquired self-sufficiency in growth signals, sustaining proliferative signalling, evading apoptosis, and resisting cell death that comprise major biological capabilities of carcinogenesis (19, 20). Other alterations associated with the cellular architecture, communication, and epithelium proliferation may reflect potential for epithelial-to-mesenchymal transition (EMT), which contributes to increased migratory potential of cancer cells and UCEC aggressiveness (21).

Furthermore, we selected a set of 28 genes from the turquoise module which mostly contributed to clear dimensional partitioning of UCEC patients demonstrating distinct molecular profiles of mixed grade 1 and grade 2 vs. grade 3 and simultaneously endometrioid endometrial adenocarcinoma vs. mixed group consisting of endometrioid, serous, and mixed endometrial adenocarcinoma patients (Figures 1C,D, 2C,D). To date, relevance of several of these genes was demonstrated in different studies on endometrial cancer (Table 2), however, of the greatest interest are genes which to our best knowledge have not been yet described elsewhere including *XPO1O, SASS6S, NUP1P07, UBXN2NB22B, NOL1L1, DPM1M, COMMD2D, MED3D0, GINS4S, CENPO, NIF3Fl33l1l, LRCC4C2, AFG3GL33L2L, DDX2X7, PPIB, LMF1F*, and *ZSWIM7MM7*.

**Table 2:** Literature review of marker gene relevance.

**Gene name**	**Expression profile FAV vs. UNFAV**	**Significance**	**Reference**
*ADNP*	Up	pharmacologically inducible repressor of Wnt signalling; silencing of *ADNP* expression caused increased migration, invasion and proliferation in colorectal cancer xenografts *in vivo*	(22)
*MSH2*	Up	disruption of MMR system was associated with an increased risk of endometrial cancer; carriers of germline mutations comprise a group of higher risk to develop endometrial cancer	(23)
*CSE1L*	Up	overexpression found in endometrial cancer associated with poor overall survival; depletion of *CSE1L* enhances apoptosis and decreases migratory potential	(24)
*PNO1*	Up	identified as a driver gene in cervical squamous cell carcinoma	(25)
*ATAD2*	Up	overexpression associated with development of aggressive endometrial cancer through enrichment of B-MYB translational signature	(26)
*TTK*	Up	inhibition of tumour growth through TTK inhibitors in CTNNB1-mutants *in vitro*	(27)
*KIF2C*	Up	considered as hub gene in female tumours including endometrial cancer	(28)
*XPO5*	Up	inactivation disrupts export of precursor microRNAs from the nucleus to the cytosol; re-expression of *XPO5* wild type shows suppressor properties in endometrial cancer	(29)
*HDGF*	Up	high nuclear expression correlates with endometrial cancer progression, although was not considered as an independent predictor	(30)
*CLDN6*	Up	component of tight junctions in epithelial cells, crucial for maintaining cell integrity; deregulation of *CLDN6* has been correlated with cancer progression and metastasis; considered as a tumour suppressor	(31, 32)

Additionally, these genes have shown potential towards prediction of UCEC prognosis independently of histology or grade with sensitivity over 70% and specificity exceeding 85%, thus enabling to determine a group of patients with poor outcome (Figure 4). Interestingly, the prognostic potential was confirmed independently of any clinicopathological parameters through survival analysis with Kaplan-Meier plots. It showed that both OS and DFS were differentiated much more accurately that with standard grade classification (Table 1, Figure 5). Finally, through the analysis of functional networks of Wnt downstream targets we identified several biological processes including, among others, Hedgehog, ErbB, VEGF signalling, and adhesion-associated processes considered as crucial in endometrial tumourigenesis that differed between novel molecular UCEC subgroups (Figure 6). Furthermore, we compared novel UCEC subgroups regarding occurrence of mutations and CNVs affecting major player of Wnt pathway (Supplementary Table 1) and found predominance of *APC, CTNNB1B, LRP5P*, and *TCF4F* mutations in the group endometrioid endometrial cancer cases as well as MYC copy number alteration in the group of endometrioid, mixed and serous endometrial cancer cases. These genes are well-known either tumour suppressors or oncogenes, nevertheless in this case they remain of unknown clinical significance. These findings confirmed biological significance of the previously chosen set of genes correlating with unfavourable prognosis and provided insight into mechanisms of UCEC progression.

In summary, the presented analysis showed that current histological classification of UCEC into endometrioid, mixed, and serous endometrial adenocarcinoma is not adequate. It seems that there is a group of endometrioid cancer cases which molecularly resembles mixed/serous group. This may explain the existence of clinically observed group that despite favourable diagnosis such as the mildest endometrioid UCEC type yet presents failure in treatment and aggressive course of the disease. Our findings thus highlight a significant role of Wnt signalling in endometrial tissue and indicate that worse outcome may result from alterations in the signalling which affect its downstream effectors involved in many cellular processes. Moreover, we showed that despite an optimistic prognosis some patients experience very adverse course of endometrioid endometrial cancer resulting from the serous genetic profile of Wnt targets. We therefore proposed the set of 28 genes that allow to predict UCEC clinical outcome, which makes it a highly applicable and potentially useful tool of high clinical value. Finally, this study revealed that experimental validation of such phenomenon should be of the greatest research attention since there is no available independent cohort that allow to compare the results.

## Materials and Methods

### Input Data Acquisition and Preparation

For purposes of this study, we acquired data from The Cancer Genome Atlas Uterine Corpus Endometrial Carcinoma (TCGA-UCEC data status of 28th Jan 2016) comprising RNAseq expression profiles of 20502 genes (level 3 RNAseqV2V, RSEM normalized) and corresponding clinical data of UCEC patients. Furthermore, both data types were combined followed by exclusion of patients with missing expression/clinical information that resulted in a total of 370 patients qualified for further analyses. The detailed characteristics of UCEC patients are shown in Supplementary Table 2.

All other methods regarding biospecimen procurement, isolation and sequencing of RNA are previously described by The Cancer Genome Atlas Research Network (33).

### Selection of Wnt Downstream Effectors

Targets of Wnt signalling pathway transcription factors were selected through the GTRD database (Gene Transcription Regulation database) offering collection of uniformly processed ChIP—seq data to identify transcription factor binding sites for human (34). We focused on well-known Wnt transcription factors belonging to TCF/LEF families such as *LEF1F, TCF3F*, and *TCF4F* (35) that could be found in GTRD. Finally, the input list included 2,571 genes that were recognized as Wnt downstream effectors.

### Weighted Gene Co-expression Network Analysis (WGCNA)

Module detection was conducted through applying the WGCNA algorithm available as a collection of R functions enabling the performance of weighted correlation network analysis. In brief, for each gene pair the Pearson's correlation was computed followed by the transformation into adjacency matrix using the soft-thresholding approach (power = 7) with the scale-free topology index (*R*^2^) > 0.9 (Supplementary Figures 5, 6). Subsequently, the topological overlap matrix (TOM) was constructed based on overlap between pairs of interconnected genes, which afterwards served as the input for hierarchical clustering (*hclust* function, “average” agglomeration method) and module detection by applying the dynamicTreeCut algorithm with a minimum module size of 30 genes. Detected modules were related to clinical traits of interest (histological subtype of UCEC, grade) by correlating module eigengenes (MEs) with external traits. In addition, gene significance (GS) for each module was determined as a linear regression *p-*value transformed by log10 between gene expression and clinical trait of interest. Finally, module significance (MS) was determined as average GS for all genes included in the module. Heatmaps visualizing expression profiles within particular modules were prepared with *gplots* R package (*heatmap.2* function, row clustering according to the Pearson's distance metric and “complete” agglomeration method).

### Dimensional Partitioning of UCEC Individuals—Principle Component Analysis (PCA) and Multiple Factor Analysis (MFA)

Dimensional grouping of UCEC patients according to a set of selected genes to determine the relevance of histological subtype and grade of UCEC was performed using Principle Component Analysis (PCA). Primary PCA involved 492 genes of turquoise module as active variables with colouring of individuals regarding grade and histological subtype. Subsequently, Multiple Factor Analysis (MFA) was conducted to determine the contribution of particular genes into partitioning of individuals across first and second dimensions with the turquoise module gene set as an active variable and clinical traits as supplementary variables (explanatory variables do not participate in creating dimensions in an active fashion, they are visible only for explanatory purposes). By applying the *fviz_contrib* function we selected 28 genes, whose contribution to PC1C was found above the reference line (corresponds to the expected value if the contributions of all active variables were equal, thus variables above the reference line are considered as significantly contributing to a given dimension) followed by the second PCA involving the selected set of genes. All analyses were performed using *FactoMineR* and *factoextra* R packages offering a wide range of functions dedicated for multivariate exploratory data analyses and result visualization.

### Building the Binomial Model of Logistic Regression and Receiver Operating Characteristic (ROC) Curve

To determine a predictive value of selected 28 genes, we built a binomial model of logistic regression by fitting generalized model that was specified by giving a symbolic description of identified molecular subgroups 0–253 patients with endometrioid endometrial adenocarcinoma and 1–117 patients with endometrioid, mixed, and serous endometrial adenocarcinoma) with regards to the gene set followed by prediction from the results of model fitting. Subsequently, the predictive potential of the novel marker was evaluated with the receiver operating characteristic (ROC) curve in the context of patient prognosis regarding clinical characteristics and aggressiveness of UCEC. The model was built with the *glm* R function with *prediction* from *glm*. ROC was plotted by employing *pROC* R package. Survival analysis showing predictive potential of novel marker independently of clinicopathological parameters was performed with univariate Cox regression model in R (*coxph* function). Kaplan-Meier plots were drawn with *ggplot2t* and *survmisc* R packages.

### Functional Network of Differentially Expressed Wnt Downstream Effectors Corresponding to Molecular Subtypes of UCEC—Network Analyst

NetworkAnalyst is a freely available tool allowing to perform integrative analysis of gene expression data with visual exploration of resulting functional networks (36). Hereby we used Wnt effector list as a whole (2,574 genes) with patients labelled according to the classification of their molecular profile regarding UCEC subtype (253 endometrioid endometrial adenocarcinomas vs. 117 endometrioid, mixed and serous endometrial adenocarcinomas). The analysis was performed with default settings, auto-scaling of RNAseq counts and the FDR cutoff set as 0.05. Network analysis was conducted with protein-protein interactions method, STRING confidence score cutoff set as 900 with requirement of experimental evidence and zero-order network. Functions of identified genes were defined with KEGG database.

### Mutation/Copy Number Variation Analysis

Additionally, we performed analysis of mutations and copy number variation (CNV) in UCEC patients according to novel molecular subgroups. Mutations/CNVs were obtained from cBioPortal repository (37, 38) for 42 major Wnt pathway members and compared between groups with *prop.test* function in R environment. Detailed list of analysed genes is available as Supplementary Table 1.

## Data Availability

The datasets analysed for this study can be found in the Broad Institute TCGA GDAC Firehose Repository (https://gdac.broadinstitute.org/).

## Author Contributions

EP: conceptualization and supervision. DJ and IB: data curation. KK: formal analysis. KK and MO: investigation and writing–original draft. MO: methodology. AB: project administration. DJ and IB: resources. MO: software. KK, DJ, and IB: visualization. AB and EP: writing–review and editing.

### Conflict of Interest Statement

The authors declare that the research was conducted in the absence of any commercial or financial relationships that could be construed as a potential conflict of interest.

## References

[1] Cancer Research UK Uterine Cancer Incidence Statistics. (2018). Available online at: https://www.cancerresearchuk.org/health-professional/cancer-statistics/statistics-by-cancer-type/uterine-cancer/ (accessed September 25, 2018).

[2] Doll A Abal M Rigau M Monge M Gonzalez M Demajo S. Novel molecular profiles of endometrial cancer-new light through old windows. J Steroid Biochem Mol Biol. (2008) 108:221–9. 10.1016/j.jsbmb.2007.09.020 18061438

[3] Merritt MA Cramer DW. Molecular pathogenesis of endometrial and ovarian cancer. Cancer Biomark Sect Dis Markers. (2010) 9:287–305. 10.3233/CBM-20111-0167 22112481 PMC3822435

[4] Bokhman JV. Two pathogenetic types of endometrial carcinoma. Gynecol Oncol. (1983) 15:10–17. 6822361 10.1016/0090-8258(83)90111-7

[5] Okuda T Sekizawa A Purwosunu Y Nagatsuka M Morioka M Hayashi M. Genetics of endometrial cancers. Obstet Gynecol Int. (2010) 2010:984013. 10.1155/2010/984013 20396392 PMC2852605

[6] O'Hara AJ Bell DW. The genomics and genetics of endometrial cancer. Adv Genomics Genet. (2012) 2012:33–47. 10.2147/AGG.S2S8953 22888282 PMC3415201

[7] Kandoth C Schultz N Cherniack AD Akbani R Liu Y Shen H. Integrated genomic characterization of endometrial carcinoma. Nature. (2013) 497:67–73. 10.1038/nature1e2113 23636398 PMC3704730

[8] Talhouk A McAlpine JN. New classification of endometrial cancers: the development and potential applications of genomic-based classification in research and clinical care. Gynecol Oncol Res Pract. (2016) 3:14. 10.1186/s4s0661-016-00355-4 27999680 PMC5154099

[9] Murali R Soslow RA Weigelt B. Classification of endometrial carcinoma: more than two types. Lancet Oncol. (2014) 15:e2e68–278. 10.1016/S1S470-2045(13)705911-6 24872110

[10] Hussein YR Weigelt B Levine DA Schoolmeester JK Dao LN Balzer BL. Clinicopathological analysis of endometrial carcinomas harboring somatic POLE exonuclease domain mutations. Mod Pathol. (2015) 28:505–14. 10.1038/modpathol.2014.143 25394778

[11] Katayama S Ashizawa K Fukuhara T Hiroyasu M Tsuzuki Y Tatemoto H. Differential expression patterns of Wnt and beta-catenin/TCF target genes in the uterus of immature female rats exposed to 17a77alpha-ethynyl estradiol. Toxicol Sci. (2006) 91:419–30. 10.1093/toxsci/kfj1j67 16551644

[12] Wagner J Lehmann L. Estrogens modulate the gene expression of Wnt-7a77a in cultured endometrial adenocarcinoma cells. Mol Nutr Food Res. (2006) 50:368–72. 10.1002/mnfr.200500215 16534752

[13] Fritah A Redeuilh G Sabbah M. Molecular cloning and characterization of the human WISP-2/CCN5N gene promoter reveal its upregulation by oestrogens. J Endocrinol. (2006) 191:613–24. 10.1677/joe.1.07009 17170219

[14] Siegel RL Miller KD Jemal A Cancer Statistics 2017. CA Cancer J Clin. (2017) 67:7–30. 10.3322/caac.21387 28055103

[15] American Cancer Society. Endometrial Cancer. (2018). Available online at: https://www.cancer.org/cancer/endometrial-cancer/detection-diagnosis-staging/survival-rates.html (accessed September 25, 2018).

[16] Moreno-Bueno G Hardisson D Sanchez C Sarrio D Cassia R Garcia-Rostan G. Abnormalities of the APC/beta-catenin pathway in endometrial cancer. Oncogene. (2002) 21:7981–90. 10.1038/sj.onc.1205924 12439748

[17] Saegusa M Okayasu I. Frequent nuclear beta-catenin accumulation and associated mutations in endometrioid-type endometrial and ovarian carcinomas with squamous differentiation. J Pathol. (2001) 194:59–67. 10.1002/path.856 11329142

[18] Shedden KA Kshirsagar MP Schwartz DR Wu R Yu H Misek DE. Histologic type, organ of origin, and Wnt pathway status: effect on gene expression in ovarian and uterine carcinomas. Clin Cancer Res. (2005) 11:2123–31. 10.1158/10788-0432.CCR-04-2061 15788657

[19] Hanahan D Weinberg RA. The hallmarks of cancer. Cell. (2000) 100:57–70. 10.1016/S0092-8674(00)81683-9 10647931

[20] Hanahan D Weinberg RA. Hallmarks of cancer: the next generation. Cell. (2011) 144:646–74. 10.1016/j.cell.2011.02.013 21376230

[21] Mirantes C Espinosa I Ferrer I Dolcet X Prat J Matias-Guiu X. Epithelial-to-mesenchymal transition and stem cells in endometrial cancer. Hum Pathol. (2013) 44:1973–81. 10.1016/j.humpath.2013.04.009 23845467

[22] Blaj C Bringmann A Schmidt EM Urbischek M Lamprecht S Frohlich T. ADNP Is a Therapeutically Inducible Repressor of WNT Signaling in Colorectal Cancer. Clin Cancer Res. (2017) 23:2769–80. 10.1158/10788-0432.CCR-16-1604 27903678

[23] Beiner ME Rosen B Fyles A Harley I Pal T Siminovitch K. Endometrial cancer risk is associated with variants of the mismatch repair genes MLH1H and MSH2HH2. Cancer Epidemiol Biomark Prev Publ Am Assoc Cancer Res Cosponsored Am Soc Prev Oncol. (2006) 15:1636–40. 10.1158/10555-9965.EPI-06-0257 16985024

[24] Zhan C Zhang X Pang D High expression of CSE1L is associated with poor prognosis in breast cancer. Int J Clin Exp Pathol. (2016) 9:11788–94. Available online at: http://www.ijcep.com/files/ijcep0026227.pdf

[25] Wang M Li L Liu J Wang J. A gene interaction networkbased method to measure the common and heterogeneous mechanisms of gynecological cancer. Mol Med Rep. (2018) 18:230–42. 10.3892/mmr.2018.8961 29749503 PMC6059674

[26] Krakstad C Tangen IL Hoivik EA Halle MK Berg A Werner HM. ATAD2 overexpression links to enrichment of B-MYB-translational signatures and development of aggressive endometrial carcinoma. Oncotarget. (2015) 6:28440–52. 10.18632/oncotarget.4955 26308378 PMC4695070

[27] Zaman GJR de Roos JADM Libouban MAA Prinsen MBW de Man J Buijsman RC. TTK Inhibitors as a Targeted Therapy for CTNNB1 (beta-catenin) Mutant Cancers. Mol Cancer Ther. (2017) 16:2609–17. 10.1158/15355-7163.MCT-17-0342 28751540

[28] Suman S Mishra A. Network analysis revealed aurora kinase dysregulation in five gynecological types of cancer. Oncol Lett. (2018) 15:1125–32. 10.3892/ol.2017.7368 29391900 PMC5769420

[29] Melo SA Moutinho C Ropero S Calin GA Rossi S Spizzo R. A genetic defect in exportin-5 traps precursor microRNAs in the nucleus of cancer cells. Cancer Cell. (2010) 18:303–15. 10.1016/j.ccr.2010.09.007 20951941

[30] Wang L Jiang Q Hua S Zhao M Wu Q Fu Q. High nuclear expression of HDGF correlates with disease progressionand poor prognosis in human endometrial carcinoma. Dis Markers. (2014) 2014:298795. 10.1155/2014/298795 24692842 PMC3947826

[31] Oshima T Miwa H. Gastrointestinal mucosal barrier function and diseases. J Gastroenterol. (2016) 51:768–78. 10.1007/s0s0535-016-1207-z 27048502

[32] Yang M Li Y Shen X Ruan Y Lu Y Jin X. CLDN6 promotes chemoresistance through GSTP1P in human breast cancer. J Exp Clin Cancer Res CR. (2017) 36:157. 10.1186/s1s3046-017-06277-9 29116019 PMC5678781

[33] Cancer Genome Atlas Research Network Albert Einstein College of Medicine Analytical Biological Services Barretos Cancer Hospital Baylor College of Medicine Beckman Research Institute of City of Hope Integrated genomic and molecular characterization of cervical cancer. Nature. (2017) 543:378–84. 10.1038/nature2e1386 28112728 PMC5354998

[34] Yevshin I Sharipov R Valeev T Kel A Kolpakov F. GTRD: a database of transcription factor binding sites identified by ChIP-seq experiments. Nucleic Acids Res. (2017) 45:D6D1–7. 10.1093/nar/gkw9w51 27924024 PMC5210645

[35] Brantjes H Barker N van Es J Clevers H. TCF: lady justice casting the final verdict on the outcome of Wnt signalling. Biol Chem. (2002) 383:255–61. 10.1515/BC.2002.027 11934263

[36] Xia J Gill EE Hancock RE. NetworkAnalyst for statistical, visual and network-based meta-analysis of gene expression data. Nat Protoc. (2015) 10:823–44. 10.1038/nprot.2015.052 25950236

[37] Cerami E Gao J Dogrusoz U Gross BE Sumer SO Aksoy BA. The cBio cancer genomics portal: an open platform for exploring multidimensional cancer genomics data. Cancer Discov. (2012) 2:401–4. 10.1158/21599-8290.CD-12-0095 22588877 PMC3956037

[38] Gao J Aksoy BA Dogrusoz U Dresdner G Gross B Sumer SO. Integrative analysis of complex cancer genomics and clinical profiles using the cBioPortal. Sci Signal. (2013) 6:pl1. 10.1126/scisignal.2004088 23550210 PMC4160307

