# Supplementary material for: A Novel Set of WNT Pathway Effectors as a Predictive Marker of Uterine Corpus Endometrial Carcinoma–Study Based on Weighted Co-expression Matrices
Source: Front Oncol. 2019 May 10;9:360. doi: 10.3389/fonc.2019.00360 (PMC6524344; doi:10.3389/fonc.2019.00360)
Supplement: Supplementary file 1 [file Data_Sheet_1.docx]

Supplementary Material


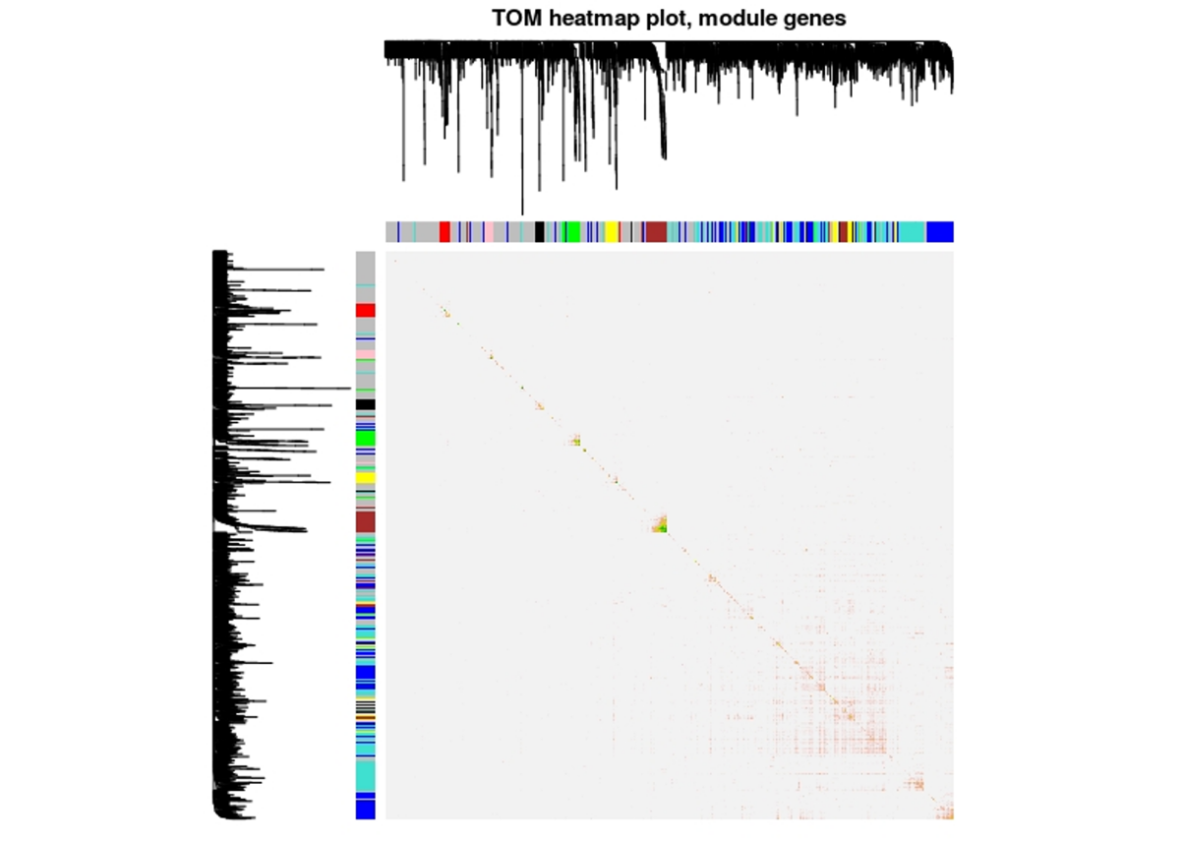


**Supplementary Figure 1.** The results of WGCNA analysis presenting topological overlap matrix plot (TOMplot) with hierarchical clustering tree based on topological overlap dissimilarity. Genes are distributed in rows and columns colored according to module of origin.


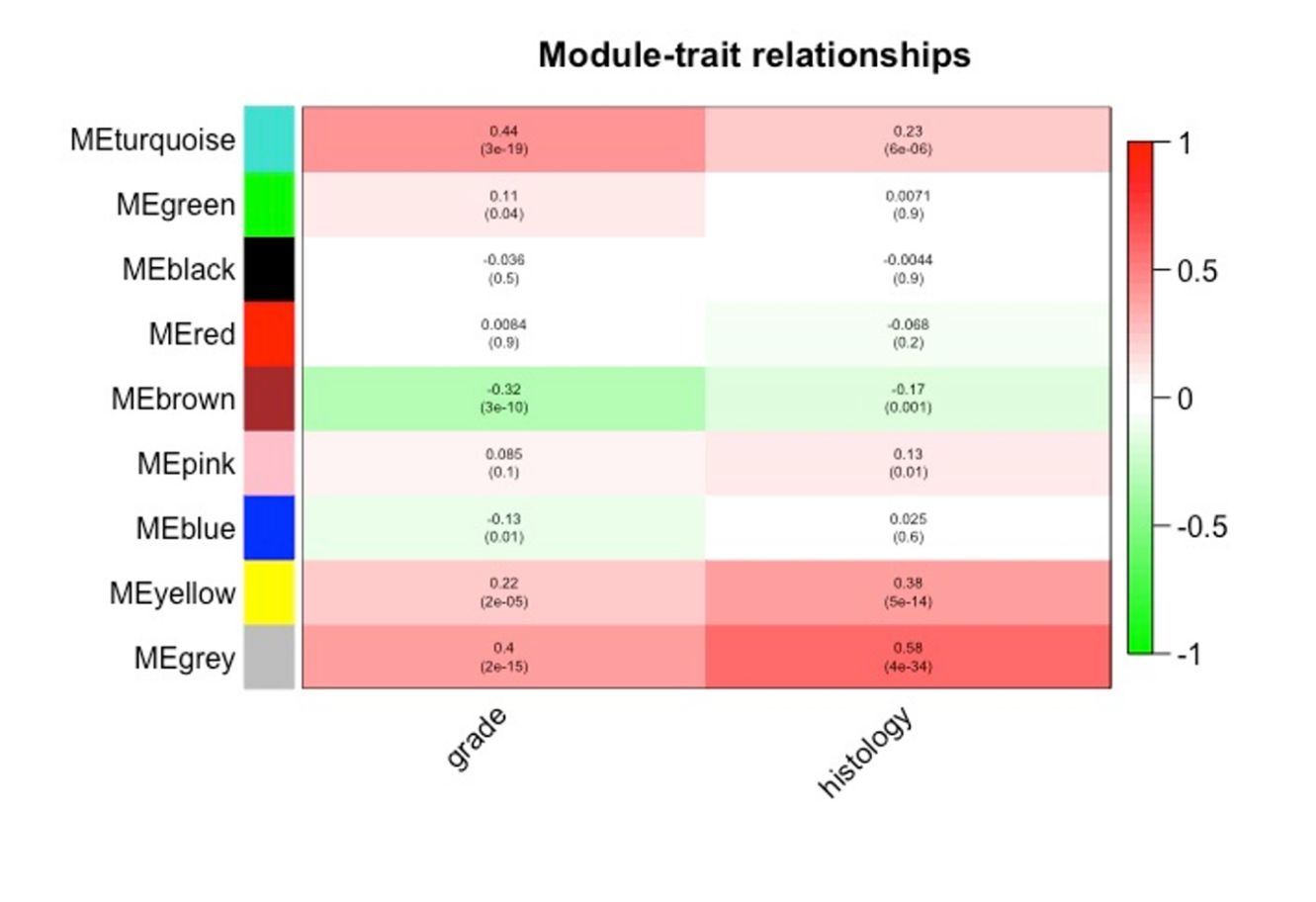


**Supplementary Figure 2.** The results of WGCNA analysis presenting module-trait relationship plot showing correlation between clinical traits of interest (grade, histology) and particular modules. Of the considered modules, turquoise showed the highest significant correlation with grade (r=0.44), whereas yellow correlated with histology of UCEC (r=0.38).


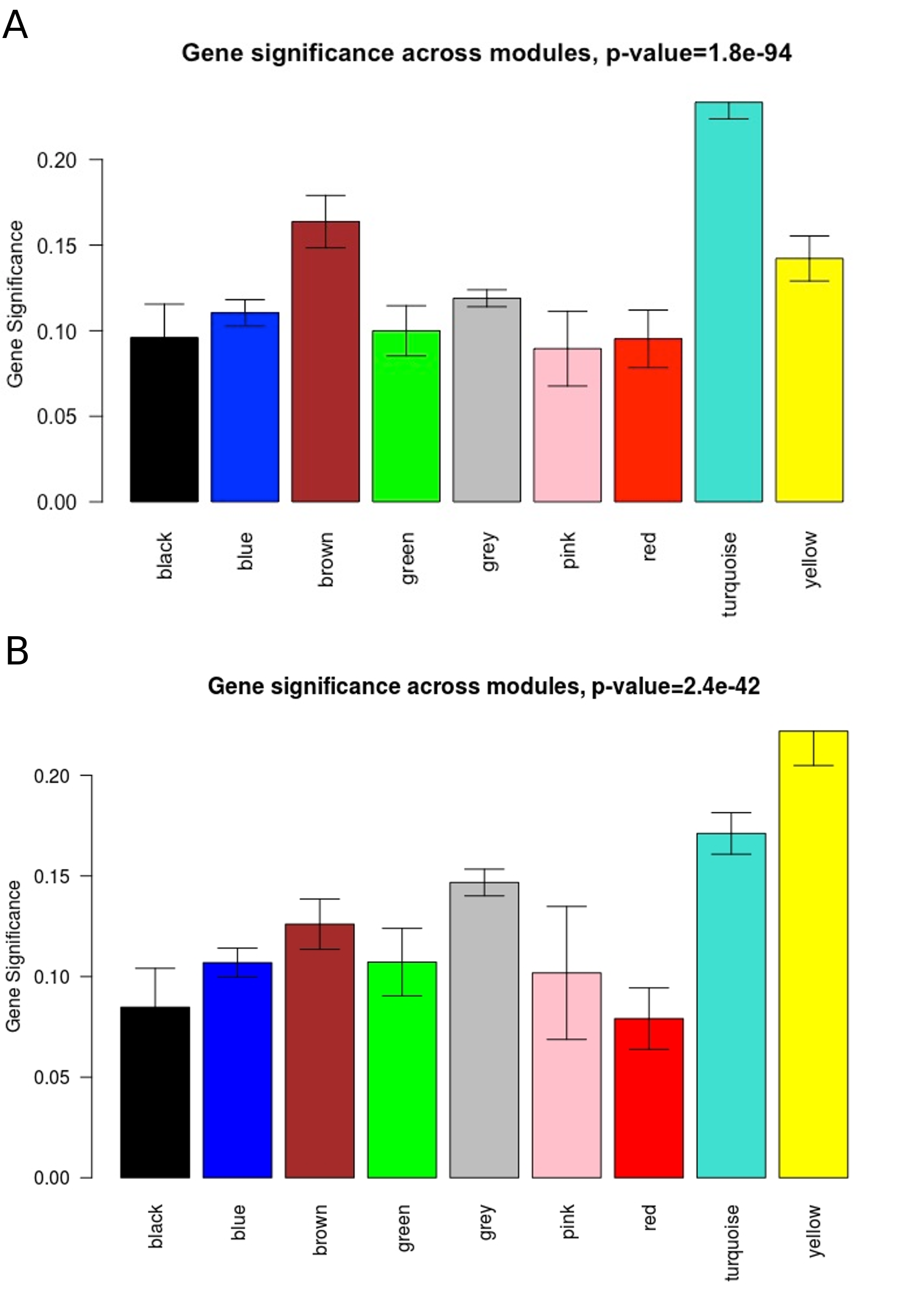


**Supplementary Figure 3.** Plots of module significance defined as mean gene significance across all genes in the module. Regarding A) grade turquoise module was the most promising and B) histology yellow module was the most promising.


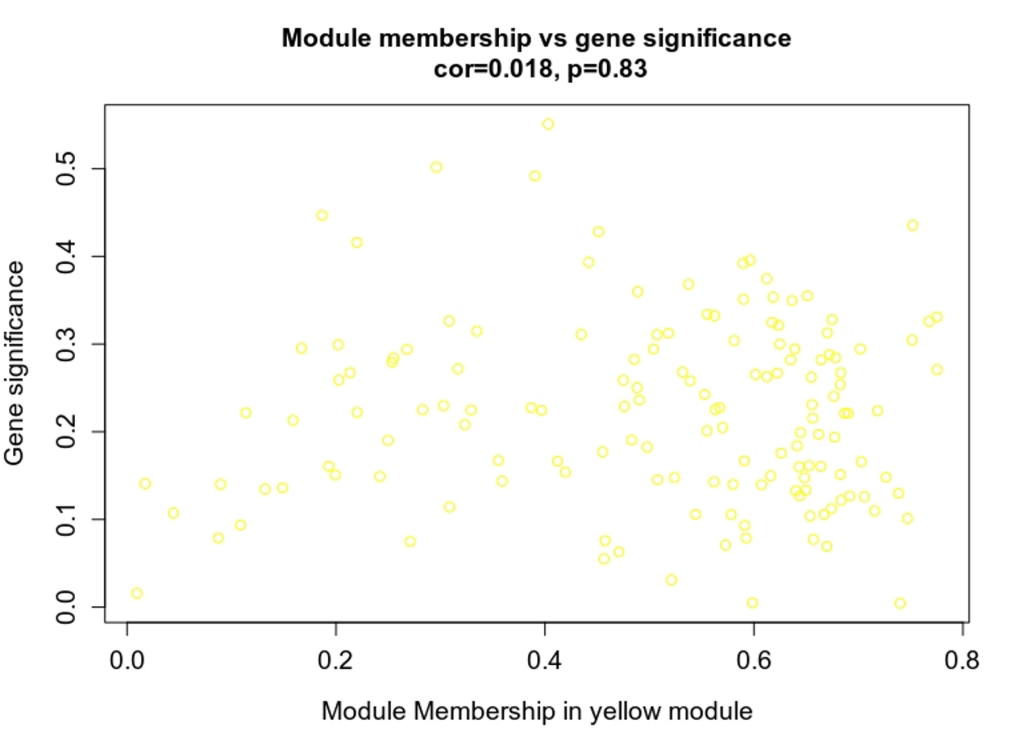


**Supplementary Figure 4.** Scatterplot of module membership vs gene significance showing insufficient association (p=0.83).


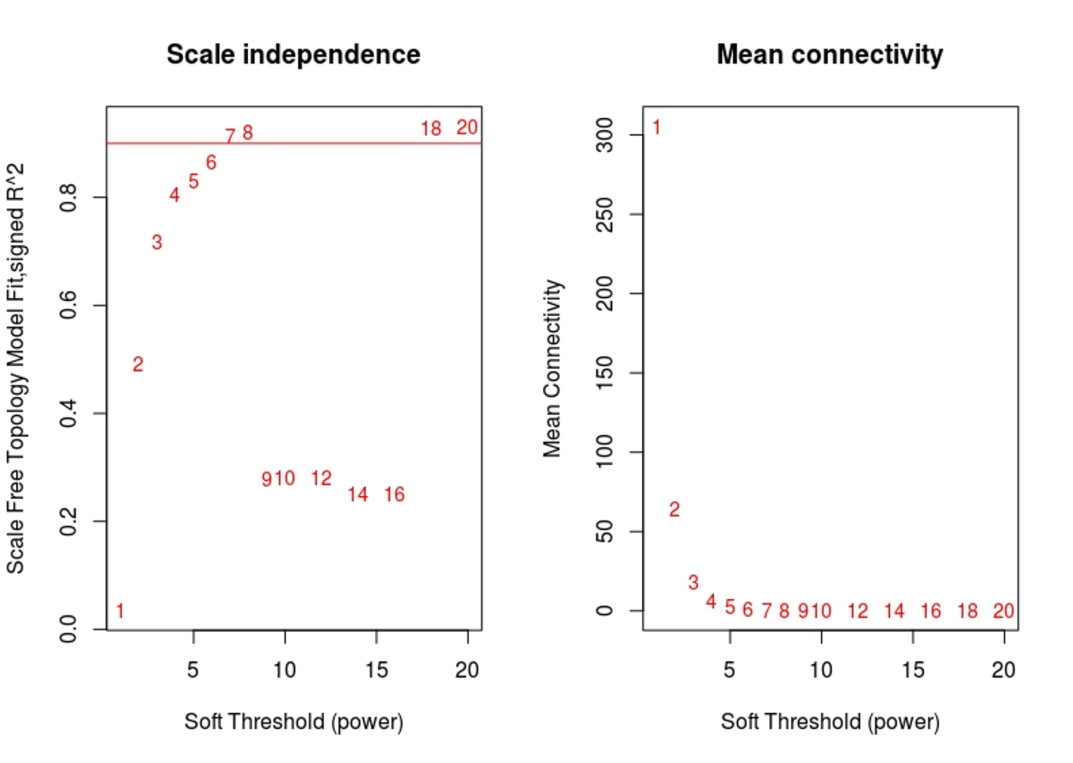


**Supplementary Figure 5.** Soft-thresholding screening plot. Left: the scale-free fit index versus soft-thresholding power. Right: the mean connectivity versus soft-thresholding power.


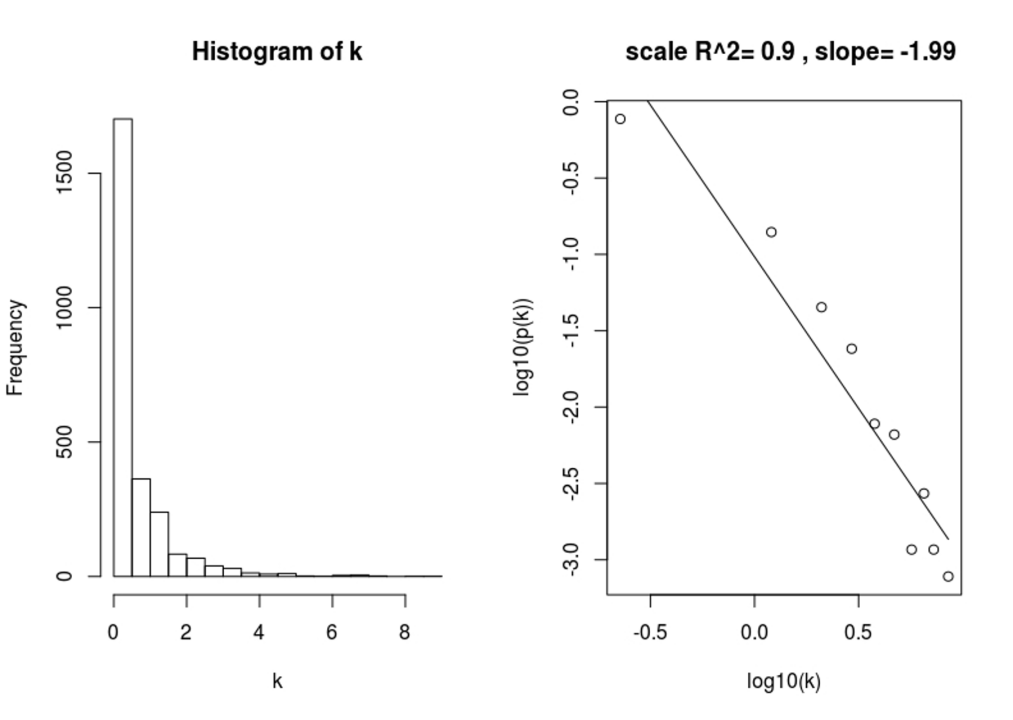


**Supplementary Figure 6.** Scale-free plot being a visual check of scale-free topology of WGCNA.

**Supplementary Table 1.** Detailed results of mutation/CNV analysis of UCEC patients.

| GENE | MUTATIONS | | | CNV | | |
| --- | --- | --- | --- | --- | --- | --- |
|  | A* | B* | p-value | A* | B* | p-value |
| ***APC*** | **26** | **2** | **0.007** | 1 | 3 | 0.181 |
| *AXIN1* | 9 | - | 0.089 | 1 | 3 | 0.181 |
| *AXIN2* | 9 | - | 0.089 | 3 | 2 | - |
| ***CTNNB1*** | **70** | **2** | **<0.001** | **-** | **3** | **0.053** |
| *DVL1* | 1 | 1 | - | - | - | - |
| *DVL2* | 9 | - | 0.09 | - | - | - |
| *FZD5* | 2 | - | 0.84 | - | - | - |
| *FZD7* | 3 | - | 0.575 | - | - | - |
| *FZD8* | 1 | - | - | - | - | - |
| *GSK3B* | 9 | - | 0.089 | 2 | 3 | 0.374 |
| *LEF1* | 8 | - | 0.119 | - | 2 | 0.186 |
| ***LRP5*** | **11** | **-** | **0.049** | 4 | 6 | 0.107 |
| *LRP6* | 17 | 3 | 0.162 | - | - | - |
| ***MYC*** | 7 | 1 | 0.429 | **10** | **18** | **<0.001** |
| *PPARD* | 3 | - | 0.575 | - | - | - |
| *SFRP1* | 4 | - | 0.408 | - | - | - |
| *SFRP4* | 7 | 1 | 0.429 | - | - | - |
| *ROR1* | 8 | - | 0.119 | - | - | - |
| *ROR2* | 8 | 1 | 0.329 | 2 | 1 | - |
| ***TCF4*** | **12** | **-** | **0.037** | - | - | - |
| *TCF7* | 3 | - | 0.575 | - | - | - |
| *TCF7L1* | 5 | - | 0.295 | - | - | - |
| *TCF7L2* | 8 | 1 | 0.329 | 1 | 2 | 0.491 |
| *WNT1* | - | - | - | - | - | - |
| *WNT2* | 3 | - | 0.575 | - | - | - |
| *WNT2B* | 6 | 1 | 0.558 | - | - | - |
| *WNT3* | 3 | - | 0.575 | - | - | - |
| *WNT3A* | - | - | - | - | - | - |
| *WNT4* | - | - | - | - | - | - |
| *WNT5A* | 4 | 1 | 0.937 | - | - | - |
| *WNT5B* | 3 | - | 0.575 | - | - | - |
| *WNT6* | - | - | - | - | - | - |
| *WNT7A* | 2 | 1 | - | - | - | - |
| *WNT7B* | 1 | 1 | - | - | - | - |
| *WNT8A* | 1 | 1 | - | - | - | - |
| *WNT8B* | 5 | - | 0.295 | - | - | - |
| *WNT9A* | 4 | - | 0.408 | - | - | - |
| *WNT9B* | - | - | - | - | - | - |
| *WNT10A* | 2 | - | 0.84 | - | - | - |
| *WNT10B* | - | - | - | - | - | - |
| *WNT11* | 4 | - | 0.408 | - | - | - |
| *WNT16* | 7 | - | 0.159 | - | - | - |

*) A - group of 253 UCEC patients including endometrioid endometrial cancer cases; B - group of 117 UCEC patients including endometrioid, mixed and serous endometrial cancer cases

**Supplementary Table 2.** UCEC patients’ characteristics.

| Clinical characteristics | TOTAL (360) | % |
| --- | --- | --- |
| Age at diagnosis [y]  - median age (range) | 63 (31 - 90) | |
| Birth control  - current user  - former user  - never used  - NA | 1  45  115  209 | 0.3  12.2  31.1  56.5 |
| Colorectal cancer in interview  - no  - yes  - NA | 328  3  39 | 88.6  0.8  10.5 |
| Hormonal therapy  - no  - yes (current user)  - yes (former user)  - NA | 208  3  32  127 | 56.2  0.8  8.6  34.3 |
| Hypertension  - no  - yes  - NA | 133  197  13 | 35.9  53.2  3.5 |
| Cancer status  - tumour free  - with tumour  - NA | 302  55  13 | 81.6  14.9  3.5 |
| Diabetes  - no  - yes  - NA | 220  88  62 | 59.5  23.8  16.7 |
| Pregnancies  - 0  - 1  - 2  - 3  - 4+  - NA | 56  45  97  54  61  57 | 15.1  12.2  26.2  14.6  16.5  15.4 |
| Prior tamoxifen administered usage  - current user  - former user  - never used  - NA | 1  6  256  107 | 0.3  1.6  69.2  28.9 |
| Radiation therapy  - no  - yes  - NA | 206  162  2 | 55.7  43.8  0.5 |
| Targeted therapy  - no  - yes  - NA | 222  120  28 | 60  32.4  7.6 |
| Vital status  - alive  - dead | 338  32 | 91.4  8.6 |
| Height [cm]  - median height (range) | 161 (130 - 181) | |
| Histological type  - endometrioid endometrial adenocarcinoma  - mixed serous and endometrioid endometrial adenocarcinoma  - serous endometrial adenocarcinoma | 303  10  57 | 81.9  2.7  15.4 |
| Neoadjuvant treatment  - no  - yes | 369  1 | 99.7  0.3 |
| Menopause status  - indeterminate (neither pre- or postmenopausal)  - peri (6 - 12 months since last menstrual period)  - post (prior bilateral ovariectomy or >12 or more since the last menstrual period with no prior hysterectomy)  - pre (<6 months since the last menstrual period and no prior bilateral ovariectomy and not on oestrogen replacement)  - NA | 14  14  300  26  16 | 3.8  3.8  81.1  7  4.3 |
| Grade  - g1  - g2  - g3 | 85  100  185 | 23  27  50 |
| Race  - American Indian or Alaska native  - native Hawaiian or other pacific islander  - Asian  - white  - black or African American  - NA | 3  6  19  288  42  12 | 0.8  1.6  5.1  77.8  11.3  3.2 |
| Stage  - I  - IA  - IB  - IC  - II  - IIA  - IIB  - IIIA  - IIIB  - IIIC  - IIIC1  - IIIC2  - IV  - IVA  - IVB | 1  118  106  19  14  6  8  25  2  23  15  14  2  2  15 | 0.3  31.9  28.6  5.1  3.8  1.6  2.2  6.8  0.5  6.2  4.1  3.8  0.5  0.5  4.1 |
| Surgical approach  - minimal invasive  - open  - NA | 121  243  6 | 32.7  65.7  1.6 |
| Weight [kg]  - median weight (range) | 86 (44 - 209) | |
| Microsatellite instability  - indeterminate  - high  - low  - stable | 2  123  31  213 | 0.5  33.2  8.4  57.6 |
